# A Novel Catheter Shape-Sensing Method Based on Deep Learning with a Multi-Core Optical Fiber

**DOI:** 10.3390/s23167243

**Published:** 2023-08-18

**Authors:** Fei Han, Yanlin He, Hangwei Zhu, Kangpeng Zhou

**Affiliations:** 1 Key Laboratory of the Ministry of Education for Optoelectronic Measurement Technology and Instrument, Beijing Information Science & Technology University, Beijing 100192, China; 2021020148@bistu.edu.cn (F.H.); hangweizhu@126.com (H.Z.); pengzkp@tju.edu.cn (K.Z.); 2 Guangzhou Nansha Intelligent Photonic Sensing Research Institute, Beijing Information Science and Technology University, Beijing 511462, China; 3 School of Locomotive and Rolling Stock Engineering, Dalian Jiaotong University, Dalian 116028, China; 4 State Key Laboratory of Precision Measuring Technology and Instruments, Tianjin University, Tianjin 300072, China

**Keywords:** multi-core optical fiber, catheter shape sensing, PSO-BP neural network, temperature compensation

## Abstract

In this paper, we propose a novel shape-sensing method based on deep learning with a multi-core optical fiber for the accurate shape-sensing of catheters and guidewires. Firstly, we designed a catheter with embedded multi-core fiber containing three sensing outer cores and one temperature compensation middle core. Then, we analyzed the relationship between the central wavelength shift, the curvature of the multi-core Fiber Bragg Grating (FBG), and temperature compensation methods to establish a Particle Swarm Optimization (PSO) BP neural network-based catheter shape sensing method. Finally, experiments were conducted in both constant and variable temperature environments to validate the method. The average and maximum distance errors of the PSO-BP neural network were 0.57 and 1.33 mm, respectively, under constant temperature conditions, and 0.36 and 0.96 mm, respectively, under variable temperature conditions. This well-sensed catheter shape demonstrates the effectiveness of the shape-sensing method proposed in this paper and its potential applications in real surgical catheters and guidewire.

## 1. Introduction

Since 1990, cardiovascular disease has been a major cause of health problems, and the mortality rate from coronary heart disease is increasing annually [1]. The main cause of coronary heart disease is atherosclerosis, which leads to myocardial ischemia and, in more severe cases, sudden cardiac death [2]. Interventional surgery has become the treatment of choice for most patients as it is less invasive, involves less bleeding and faster recovery than traditional open surgery, and effectively reduces the risk of postoperative complications. The method of guiding the catheter to the target position during interventional procedures is based on medical imaging, which allows the surgeon to see the shape of the catheter in the vessel in real time and prevent damage to the vessel wall. Current medical imaging methods include fluoroscopy, ultrasound imaging, and magnetic resonance imaging (MRI) [3,4,5]. In fluoroscopy, which requires uninterrupted use during the procedure, the vascular structure is clearly seen, but the patient and surgeon are both exposed to high radiation levels. A contrast agent is also administered to the patient. Ultrasound imaging is safer than fluoroscopic imaging as it is radiation-free and can show vascular structures. However, it has a low signal-to-noise ratio and does not provide accurate information about the shape of the vessels and catheters. The main disadvantages of these three medical imaging types are the high radiation dose, low image signal-to-noise ratios and susceptibility to metal. Therefore, a method that can reduce the radiation dose and provide accurate information about catheter shape is needed.

Multi-core optical fibers have been gradually used as a sensor in the medical, construction, industrial, and aerospace fields [6,7,8,9]. The small size, ease of integration, immunity to electromagnetic interference, fast interrogation, and quasi-distributed sensing of multi-core fibers have led to widespread interest in shape sensing [10,11]. Recently, research has been conducted in the field of multi-core fiber shape sensing. Since needles displayed in MRI images are subject to signal interference from factors such as blood vessels, Battisti et al. [12] proposed a prototype needle embedded in three single-core fibers with an accuracy of 0.79 mm for the needle shape. However, the MRI images are long and susceptible to metal objects. Denasi et al. [13] proposed a catheter ultrasound image fusion fiber sensing method that achieves the position measurement of the catheter tip but not the catheter shape measurement. In 2019, Khan proposed a multi-core fiber shape-sensing method that could be applied to flexible medical devices [14]. It involved embedding four multi-core fibers into a catheter reconstructed by Frenet–Serret equations. The author’s reason for using multiple multi-core fibers was to improve the possibility of preventing sensor system failures. However, the cost was prohibitive. Fluoroscopy during endovascular aortic repair (EVAR) procedures can pose a radiation hazard to patients and surgeons. To reduce the amount of radiation, Sonja embedded a multi-core optical fiber into the catheter [15], which had 38 FBG arrays. By analyzing the factors affecting shape reconstruction, he proposed an optimized shape-sensing model with an average error of 0.35 to 1.15 mm in 3D shape reconstruction. For clinical application, Sonja integrated three electromagnetic (EM) sensors into the catheter [16] and transformed the fiber-reconstructed shape into CT space via a spatial transformation matrix. This method positioned the catheter shape in space with an average error of <0.9 mm and a maximum error of <2.3 mm in the reconstruction shape. Xuan et al. [17] proposed combining multi-core optical fiber, two EM sensors, and fluoroscopy images to measure and compensate for the twist problem. However, the EM sensor is easily affected by metal objects and the method is costly. In 2023, Li et al. [18] proposed combining polynomial fitting and neural networks for reconstructing shapes, which avoids the complex computation from wavelength data to fitted shapes. However, polynomial fitting to reconstruct shapes can cause variations in reconstructed shapes due to deviations in key position information. Sefati et al. [19] proposed a data-driven shape-sensing method that achieves the reconstruction of the manipulator shape using the distal position in a constrained environment, but neural networks are complex and computationally intensive. Xuan et al. [20] proposed an ANN-based method for the shape measurement of continuum robots. However, its neural network prediction results were unstable because the initial weights and thresholds were not determined.

In response to the problems of the aforementioned research, we propose a shape reconstruction method based on a PSO-BP neural network with a multi-core optical fiber sensing catheter. Firstly, we designed the structure of the catheter embedded in the multi-core optical fiber, then analyzed the relationship between the FBG central wavelength shift and curvature, and established a catheter shape sensing model based on PSO-BP neural network. To eliminate the effects of temperature change on the FBG wavelength shift, we studied the temperature compensation method of multi-core optical fiber temperature sensitivity. We also compared the errors of multi-core optical fiber sensing catheter shapes using different BP and PSO-BP neural networks.

This paper is organized as follows: In Section 2, we describe the structure of the catheter with embedded optical fiber sensors. In Section 3, the FBG sensing principle and the PSO-BP neural network-based catheter shape sensing method are described. In Section 4, the experimental setup and testing are described, and the results are analyzed. Section 5 presents conclusions and future works.

## 2. Design of the Catheter with Embedded FBG Sensors

**Figure 1:**
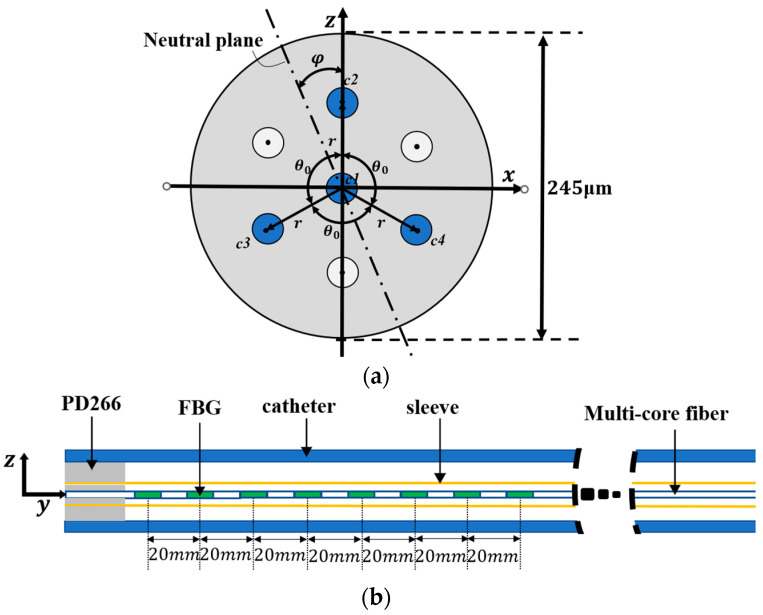
Catheter structure for implanting optical fiber sensors. (**a**) Fiber cross-section. (**b**) Catheter sensing structure.

Figure 1 shows the catheter structure for the multi-core fiber implantation, with an inner diameter of 2.06 mm and a length of 1 m. The multi-core fiber is first placed in a sleeve with an inner diameter of 900 μm and then inserted into the catheter, where they are fixed at a distance of 2 cm from the first set of fiber gratings using a DP2216 epoxy adhesive. The multi-core fiber has a diameter of 245 μm and an equal distance of 41.5 μm between adjacent cores. The sensing region of the multi-core fiber has a length of 140 mm. Each core of the multi-core fiber contains 8 gratings with a 20 mm separation between the centers of each grating. Three outer cores in the fiber are used, and the separation angle is 120°, with one core in the middle for temperature compensation. The length of each grating is 10 mm. The multi-core fiber contains eight groups of FBGs, noted as FBG1, FBG2, FBG3, FBG4, FBG5, FBG6, FBG7, FBG8. The initial center wavelengths of the eight gratings are 1530, 1534, 1538, 1542, 1546, 1550, 1554, and 1558 nm, respectively.

## 3. Multi-Core Fiber Shape Sensing Algorithm

### 3.1. FBG Sensing Principle

FBG sensors can reflect a part of the incoming light. The wavelength of the reflected light is the Bragg wavelength *λ_B_*, which is related to the grating period Λ and effective refractive index according to the mode coupling theory [21]:

(1) $$\lambda_{B}=2n_{eff}\Lambda$$

where *λ_B_* and Λ are in nm and *n_eff_* is a dimensionless number. Both strain and temperature changes due to bending or twisting affect the change in FBG reflection wavelength. The FBG wavelength shift is expressed as [22]:

(2) $$\Delta \lambda_{B}=\lambda_{B,0}((1-P_{e})\Delta \varepsilon +(\alpha_{\Lambda }+\alpha_{n})\Delta T)$$

where *λ_B_*_,0_ represents the initial central wavelength of the FBG in the unstressed condition; *P_e_* represents the photo-elastic coefficient; *α*_Λ_ and *α_n_* represent the thermal expansion coefficient and thermo-optical coefficient of the fiber, respectively; and Δ*ε* and Δ*T* represent the FBG strain change and temperature change, respectively.

A further simplification of Equation (2) is expressed as:

(3) $$\Delta \lambda_{B}=m_{\varepsilon }\Delta \varepsilon +m_{T}\Delta T$$

where *m_ε_* and *m_T_* represent the strain sensitivity coefficient and the temperature sensitivity coefficient, respectively.

The middle core c1 is located on the neutral plane, and the fiber is considered to be a pure bending model. Therefore, the strain caused by the bending or twisting of the middle core is neglected. The change in FBG wavelength shift for the four cores can be expressed as:

(4) $$\left\{\begin{array}{l} \Delta \lambda_{B1}=m_{T1}\Delta T\\ \Delta \lambda_{Bi}=m_{\varepsilon i}\Delta \varepsilon +m_{Ti}\Delta T \end{array}\right.$$

*λ_Bi_* (*i* = 2, 3, 4) represents the FBG wavelength shift of each core since each core is different. The temperature sensitivity of each core must be calculated as follows:

(5) $$m_{Ti}=k_{i}m_{T1}$$

The wavelength shift Δ*λ_i_* after temperature compensation is obtained from Equations (5) and (4):

(6) $$\Delta \lambda_{i}=\Delta \lambda_{Bi}-k_{i}\Delta \lambda_{B1}=m_{\varepsilon i}\Delta \varepsilon$$

It is possible to determine the relationship between the strain on the fiber and the distance from the fiber to the neutral plane by selecting a section of the fiber with a micro-arc length *ds* and constructing a pure bending model of the fiber while neglecting twist [23]:

(7) $$\varepsilon =\frac{ds-dl}{dl}=\frac{(r+\delta )d\theta -rd\theta }{rd\theta }=\frac{\delta }{r}=k\delta$$

where *ds* is the arc length where the fiber is located; *dl* is the neutral plane arc length; *δ* is the distance from the fiber to the neutral plane; *k* is the curvature; and *r* is the radius. The relationship between curvature and wavelength shift can be obtained as follows from Equations (2) and (7):

(8) $$k=\frac{\Delta \lambda_{B}}{\lambda_{B0}(1-P_{e})\delta }$$

The relationship between FBG strain, curvature, and orientation angle in the three cores can be derived from the cross-sectional view of the fiber in Figure 1:

(9) $$\left\{\begin{array}{l} \varepsilon_{2}=kr\sin(\varphi )+\varepsilon_{0}\\ \varepsilon_{3}=kr\sin(\varphi +\theta_{0})+\varepsilon_{0}\\ \begin{array}{l} \varepsilon_{4}=kr\sin(\varphi +2\theta_{0})+\varepsilon_{0}\\ \varphi =\varphi_{r}+\varphi_{t} \end{array} \end{array}\right.$$

where *ε_i_* (*i* = 2, 3, 4) denotes strain; *k* denotes curvature; *φ* denotes the angle between the neutral axis and the *z*-axis, which is the directional angle; *θ*_0_ denotes 120°; and *ε*_0_ denotes the strain bias due to temperature change and strain. The presence of twist in the fiber affects the angle *φ*; $\varphi_{r}$ denotes the true angle and $\varphi_{t}$ denotes the twist angle. The angle *φ* is compensated by the shape reconstruction result. *ε*_0_ is considered the same for all three cores and the curvature *k* and directional angle *φ* can be obtained by solving Equation (9).

**Figure 2:**
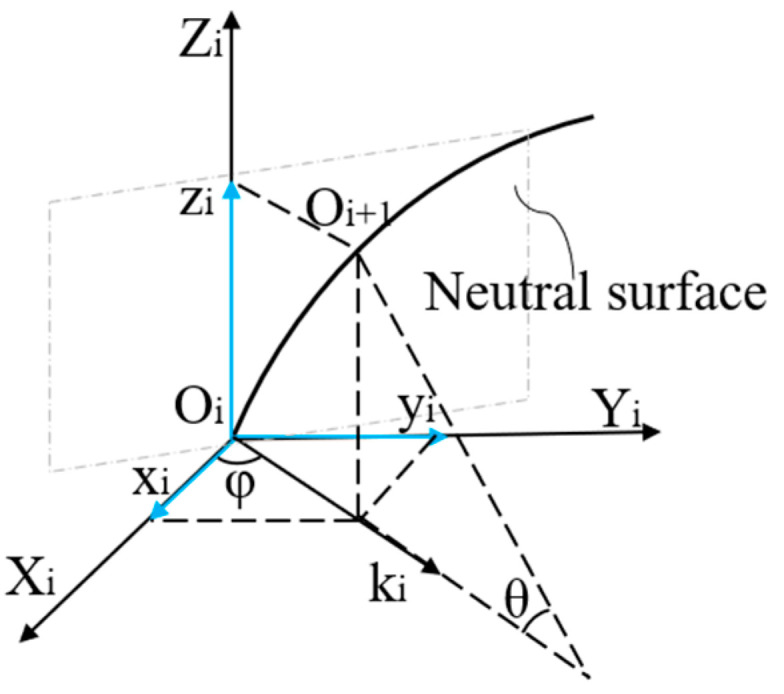
Catheter motion coordinate system.

According to differential geometry theory, we established a motion coordinate system *O_i_* (*i* = 1, 2, ..., n), as shown in Figure 2. Assuming that the curvature size of point *i* is *k_i_*, the second node is *O_i+_*_1_. The arc length between the two points is *ds* and the corresponding angle is *θ*. The coordinates of node *O_i+_*_1_ under the *O_i_* coordinate system can be deduced as follows:

(10) $$\left\{\begin{array}{l} x_{i+1}=r_{i}(1-\cos\theta_{i})\cos\varphi_{i}\\ y_{i+1}=r_{i}(1-\cos\theta_{i})\sin\varphi_{i}\\ z_{i+1}=r_{i}\sin\theta_{i} \end{array}\right.$$

The transformation matrix allows discrete points on the curve to be converted to the *O*_0_ coordinate system by the equation:

(11) $$M_{o}=M_{i}T_{i}$$

where ***M****_i_* are the coordinates of the other points on the curve and ***T****_i_* is the transformation matrix. We calculated the coordinate values of all discrete points on the curve under the origin coordinate system ***M***_0_ and reconstructed the shape of the catheter.

### 3.2. Design of the PSO-BP Neural Network

**Figure 3:**
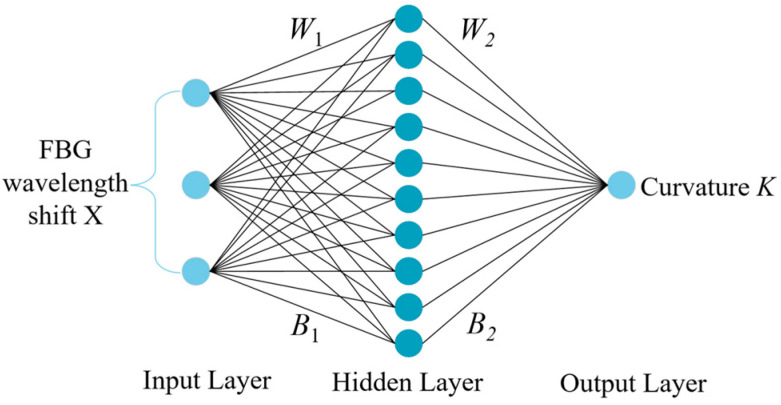
BP neural network structure.

The particle swarm optimization algorithm developed by Kennedy and Eberhart in 1995 is a method to find the global optimal solution by simulating the behavior of birds, fish, and other animals [24]. It has been widely used in the fields of architecture and aerospace [25,26]. The BP neural network is a kind of feed-forward neural network with error back propagation [27]. The network structure is shown in Figure 3, including input, hidden, and output layers. Then, the best initial weights and thresholds must be set that are suitable for this neural network [28]. The PSO algorithm can improve the prediction accuracy of the neural network by optimizing the initial weights and thresholds.

In a BP neural network, the relationship between the input and hidden layers can be expressed as [29]:

(12) $$H=f_{1}(W_{1}X+B_{1})$$

where *f*_1_ denotes the hidden layer activation function; the input layer input matrix is ***X*** = [Δ*λ*_1,_ Δ*λ*_2,_ Δ*λ*_3_]*^T^*; ***H*** = [*h*_1_, *h*_2_, *h*_3_]*^T^* is the hidden layer output matrix; and ***W***_1_, ***B***_1_ denote the input layer to hidden layer weight and threshold matrix, respectively. The size of matrix ***W***_1_ is 10 × 3; the size of matrix ***B***_1_ is 10 × 1; the size of matrix ***X*** is 3 × 353; and the size of matrix ***H*** is 10 × 353. The relationship between the hidden and output layers is expressed as:

(13) $$K=f_{2}(W_{2}H+B_{2})$$

where ***K*** denotes the output matrix; ***W***_2_ and ***B***_2_ denote the weights and threshold matrix from the hidden layer to the output layer; and *f*_2_ denotes the output layer activation function. The size of matrix ***W***_2_ is 1 × 10; the size of matrix ***B***_2_ is 1 × 1; and the size of matrix ***K*** is 1 × 353.

**Figure 4:**
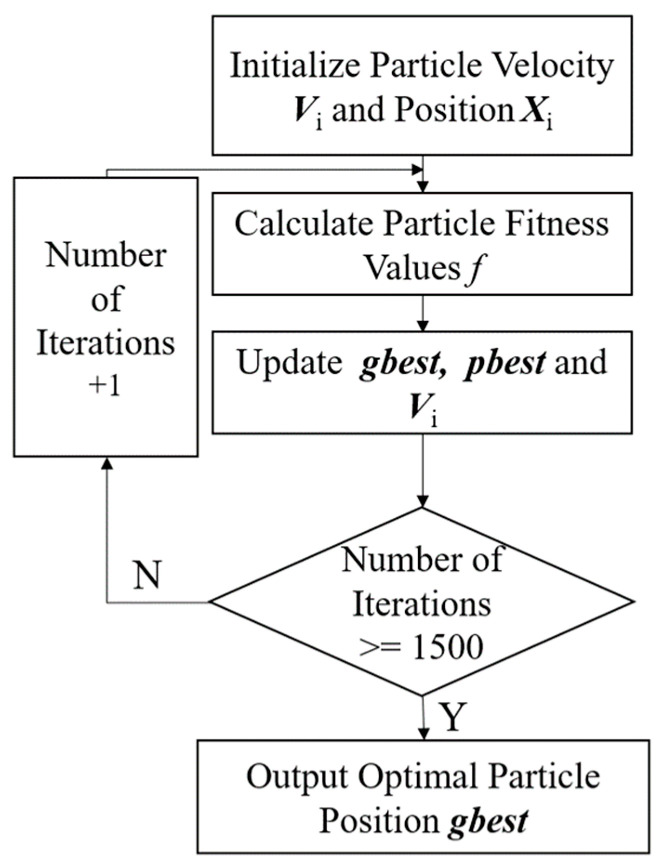
PSO optimization algorithm process.

We used the PSO algorithm to optimize the initial weights and thresholds of the neural network because BP neural networks tend to converge at local minimum values. We treated the neural network’s weight values and thresholds as particles without mass and volume, and all particles as a population with random initialization of particle positions and velocities. Figure 4 represents the PSO optimization algorithm flow.

In Figure 4, the positions and velocities of the particle swarm are first randomly initialized, and the swarm fitness value is calculated to the particle optimal position ***gbest***. The fitness function is expressed as:

(14) $$f_{3}={\sum \left(T-K\right)}^{2}$$

where ***T*** denotes the curvature truth value and ***K*** denotes the neural network prediction, which subsequently update the particle position ***X****_i_* and velocity ***V****_i_*. The size of matrix ***X****_i_* is 1 × 51 and ***V****_i_* is 1 × 51. The expression is as follows [30]:

(15) $$V_{i}^{k+1}=w^{k}V_{i}^{k}+c_{1}r_{1}(pb^{k}-X_{i}^{k})+c_{2}r_{2}(gb^{k}-X_{i}^{k})$$

(16) $$X_{i}^{k+1}=X_{i}^{k}+V_{i}^{k+1}$$

where ***pb*** denotes the global particle position, size 1 × 51; ***gb*** denotes the optimal particle position, size 1 × 51; *w* denotes the inertia weight; the maximum inertia weight and the minimum inertia weight are set to 0.9 and 0.5, respectively; *r*_1_ and *r*_2_ denote two random numbers in the range of 0–1; *c*_1_ and *c*_2_ denote the learning factors, all set to 2. The population size is set to 100 and the number of iterations is 100. The optimal particle position ***gbest*** is output after reaching the number of iterations, which is the initial weight and threshold of the BP neural network.

The input layer of the PSO-BP neural network has three neurons for the temperature-compensated FBG wavelength shift X. The output layer has one neuron for the curvature value k, the orientation angle is set to 0, and the number of neurons in the hidden layer is 10. Table 1 indicates the effect of testing different hidden layers and the number of neurons on the performance of the neural network, with the number of hidden layers ranging from one to two and the number of neurons in the hidden layer ranging from 3 to 100. The mean error and standard deviation are used to measure the prediction accuracy of the neural network. The activation function is a logsig function with the following expression:

(17) $$\log sig(x)=\frac{1}{1+e^{-x}}$$

The learning rate is set to 10^−3^. Mean square error (MSE) was used as the loss function to measure the prediction accuracy of the neural network:

(18) $$MSE=\frac{1}{N}\sum_{i=1}^{N}{\left(k_{i}-\overset{\wedge }{k_{i}}\right)}^{2}$$

where *k_i_* denotes the true value of the curvature; $\hat{k_{i}}$ denotes the predicted value of the curvature; and *N* denotes the number of samples. The catheter reconfiguration shape is represented by a point set *s* as:

(19) $$S=\left\{S_{0},\ldots ,S_{N}\right\}$$

These coordinate points are the locations of the interpolation points in the reconstructed shape. We used 32 interpolation points with a distance of 4.5 mm between the coordinate points according to the cubic spline interpolation method. The average distance error *e_avg_* and the maximum distance error *e_max_* were used to measure the reconstructed catheter shape’s accuracy. The expressions are as follows:

(20) $$e_{avg}=\frac{1}{N}\sum_{i=1}^{N}{\left\|S_{i}-S_{i}^{gt}\right\|}_{2}$$

(21) $$e_{\max}=\max({\left\|S_{0}-S_{0}^{gt}\right\|}_{2},\ldots ,{\left\|S_{N}-S_{N}^{gt}\right\|}_{2})$$

where ***S****_i_* denotes the coordinate point on the reconstruction curve and $S_{i}^{gt}$ denotes the coordinate point on the true value curve.

## 4. Experiment Testing and Analysis of Results

### 4.1. Experimental Setup

**Figure 5:**
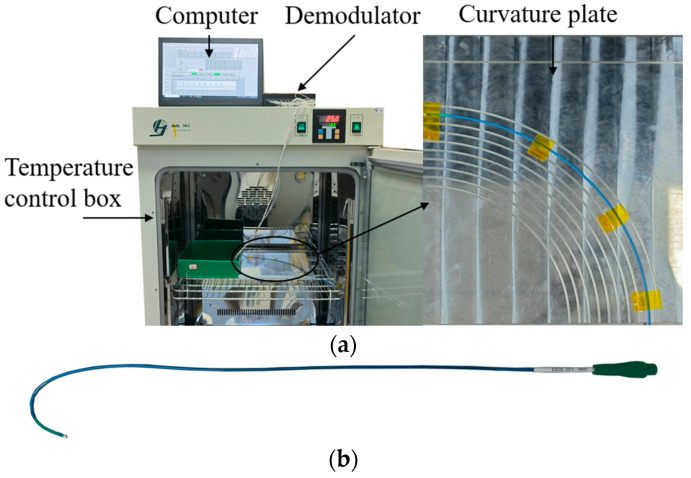
Temperature calibration system. (**a**) Temperature calibration; (**b**) catheter.

To verify the effectiveness of the PSO-BP neural network-based multi-core optical fiber sensing catheter shape reconstruction method, temperature and curvature calibration systems were built. Figure 5 shows the temperature calibration system, including a temperature control box (GNP-9270), catheter LA7EBU35 (Medtronic Inc., Minneapolis, MN, USA), demodulator, and computer. As shown in Figure 5b, the demodulator adopts the FBG demodulation module from XuFeng Photoelectric, with a measurable wavelength range of 1527–1568 nm, a resolution of 1 pm, a measurement speed of 1 Hz, and a temperature control box accuracy of 0.1 °C.

**Figure 6:**
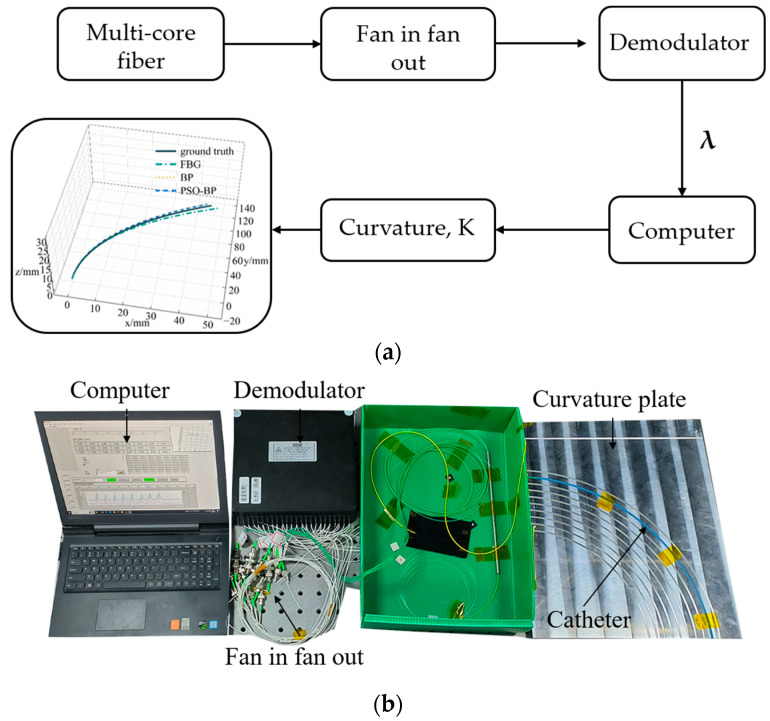
Curvature calibration system. (**a**) Flowchart; (**b**) experimental setup.

Figure 6 shows the curvature calibration system and includes a catheter, curvature calibration plate, demodulator, fan-in and fan-out device, and computer. Three curvature calibration plates made of aluminum alloy were fabricated. Each plate was engraved with 10 curvature grooves with an inner diameter of 2.4 mm to hold the catheter. The curvature ranges of the three plates were from 2.94 m^−1^ to 4.00 m^−1^, 4.17 m^−1^ to 6.67 m^−1^, and 7.14 m^−1^ to 20.00 m^−1^. The maximum curvature used in the experiment was 14.28 mm^−1^. Figure 6a shows the flow to obtain the curvature. The multi-core fiber was connected to the demodulator through a fan-in and fan-out device, and then the acquired FBG center wavelength was displayed on the computer. We calculated the curvature from the FBG center wavelength shift, and obtained the catheter shape using the shape reconstruction method.

### 4.2. Temperature Calibration and Compensation

**Figure 7:**
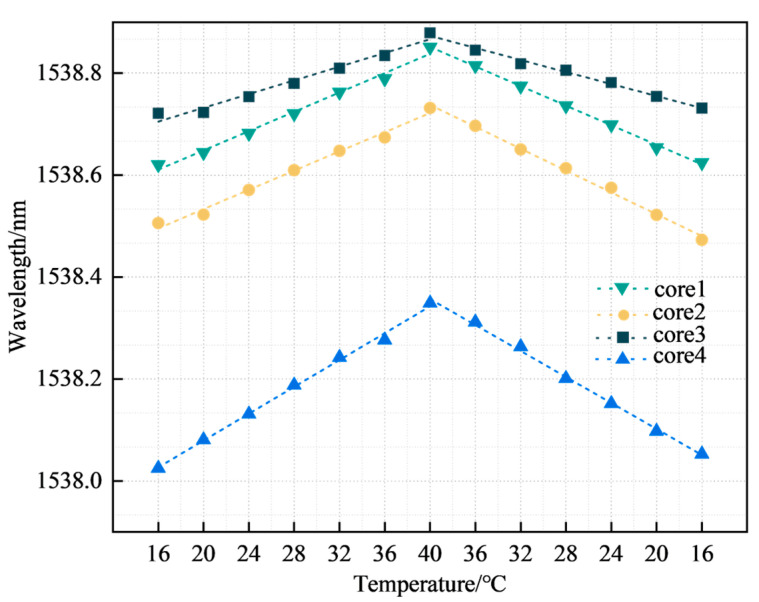
FBG temperature sensitivity.

The FBG was calibrated and compensated for temperature because multi-core fibers are affected by temperature when shape sensing. When performing cardiac surgery, the operating room temperature is typically 17 to 23 °C [31]. To simulate the temperature change of the catheter from outside the body to inside the body, the temperature change range was set between 16 °C and 40 °C. We performed wavelength acquisition of FBGs at 4 °C intervals and calculated the temperature sensitivity of all FBGs. Figure 7 represents the FBG temperature sensitivity curve in a multi-core fiber, with the third group of FBGs as an example.

**Figure 8:**
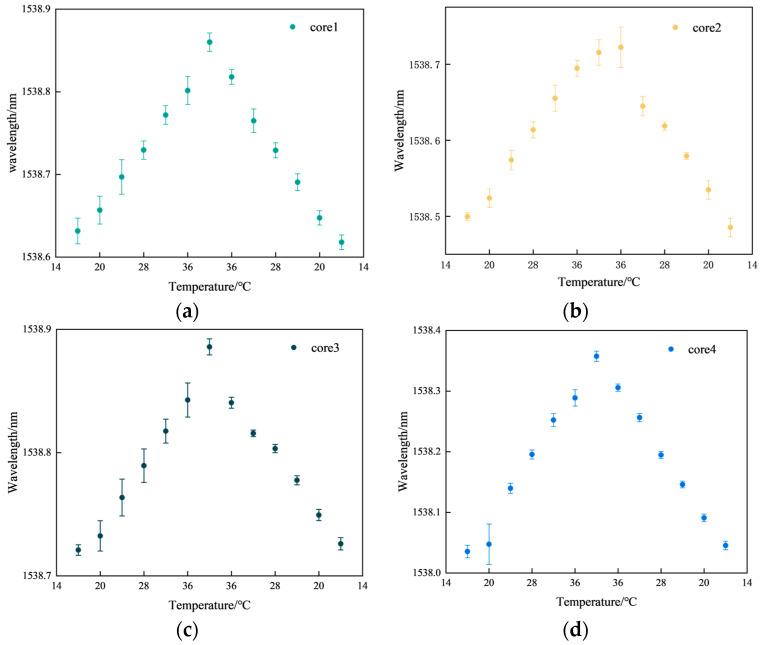
Results of temperature repeatability experiments. (**a**) Core 1; (**b**) core 2; (**c**) core 3; (**d**) core 4.

The four curves in Figure 7 represent the temperature sensitivity curves corresponding to the third group of FBGs in the four cores, with temperature sensitivities of 9.55, 10.08, 6.32, and 12.96 pm/°C respectively, all with linearities >0.97. The coefficient values of k2, k3, and k4 were 1.056, 0.662, and 1.357, respectively. The FBG sensors for cores 2, 3, and 4 were subsequently temperature compensated according to the coefficients. Figure 8 represents the temperature repeatability results of the FBG sensor in the multi-core fiber.

Figure 8a represents the FBG3 repeatability results for core 1. Figure 8b represents the FBG3 repeatability results for core 2. Figure 8c represents the FBG3 repeatability results for core 3. Figure 8d represents the FBG3 repeatability results for core 4. In Figure 8, the maximum fluctuation in the center wavelength of FBG is 0.0335 nm, and the minimum fluctuation is 0.0027 nm. Some points in the center of the wavelength fluctuation range are large when the temperature control box does not reach stable temperatures, resulting in a large number of fluctuations in the center of the FBG wavelength. However, the shape of the multi-core fiber sensing performance is not affected. The average fluctuation of core 1 is 0.0126 nm, core 2 is 0.0109 nm, core 3 is 0.0076 nm, and core 4 is 0.01 nm. According to our results, the FBG sensor has good consistency in measuring temperature and meets the experimental requirements.

### 4.3. Shape Reconstruction in a Constant Temperature Environment

For data collection, the curvature plate and other experimental equipment were placed on the horizontal table of the optical platform. Then, the catheter was placed in a straight curvature slot, left unstressed and fixed with tape, and the central wavelength and corresponding curvature of the FBG were recorded.

Keeping the catheter unrotated relative to the curvature plate and bending the catheter, the catheter was placed in the different curvature slots in sequence, with the catheter placed in the order of curvature from 2.94 to 14.28 m^−1^. The curvature placement order was then reversed and cyclically placed 63 times. The corresponding FBG central wavelength values and curvature were then recorded. A total of 504 sets of experimental data were temperature compensated and 353 sets of neural network training data and 151 sets of test data were randomly selected among the experimental data in a ratio of 7:3. Figure 8 represents the loss function curves of the training and test data after the PSO-BP neural network.

**Figure 9:**
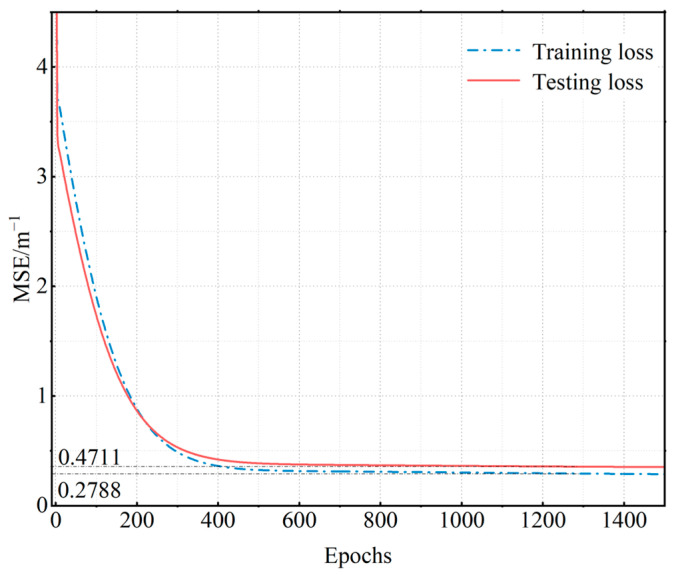
Neural network loss function curves.

Figure 9 indicates that the loss function converges after reaching 800 iterations. After 1500 iterations, the mean squared error is 0.2788 m^−1^ for the training datasets and 0.4711 m^−1^ for the testing datasets.

**Figure 10:**
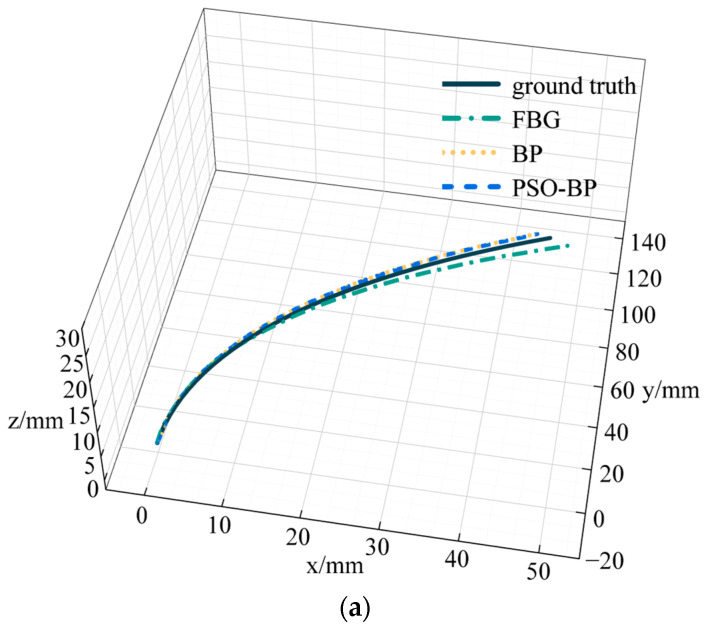

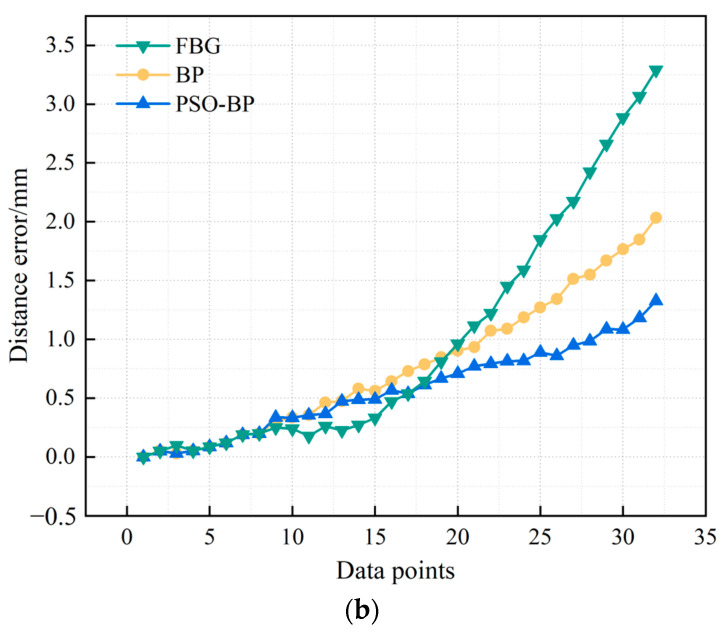
Results and errors of catheter shape reconstruction. (**a**) Shape reconstruction results; (**b**) distance error.

To verify the reliability of the PSO-BP neural network for curve reconstruction, we also selected a BP neural network for curve reconstruction. The number of neurons in the input layer of the BP neural network was 3, the number of neurons in the hidden layer was 10, and the number of neurons in the output layer was 1. We chose the logsig function and MSE for the activation function and loss function, respectively. The learning rate was $1\times 10^{-3}$. Figure 10 represents catheter shape reconstruction results for different algorithms with a curvature of 4.762 m^−1^.

FBG in Figure 10 represents the results calculated by the curve reconstruction algorithm in Section 3.1. BP and PSO-BP represent the results of curve reconstruction by different neural networks. The average distance error of the catheter shape obtained by the curve reconstruction algorithm is 0.99 mm and the maximum distance error is 3.29 mm. The average distance error of the BP neural network reconstruction of the catheter shape is 0.78 mm, and the maximum distance error is 2.03 mm. The average distance error of the PSO-BP neural network reconstruction of the catheter shape is 0.57 mm, and the maximum distance error is 1.33 mm.

### 4.4. Shape Reconstruction in a Variable Temperature Environment

**Figure 11:**
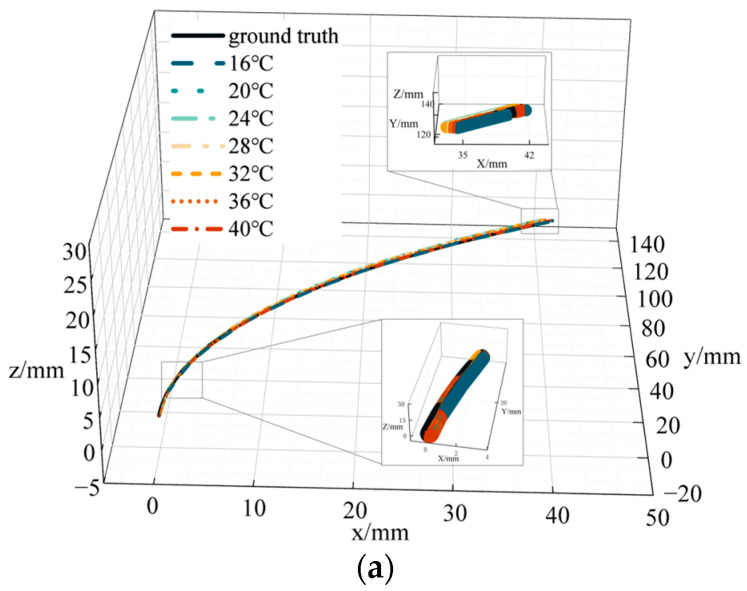

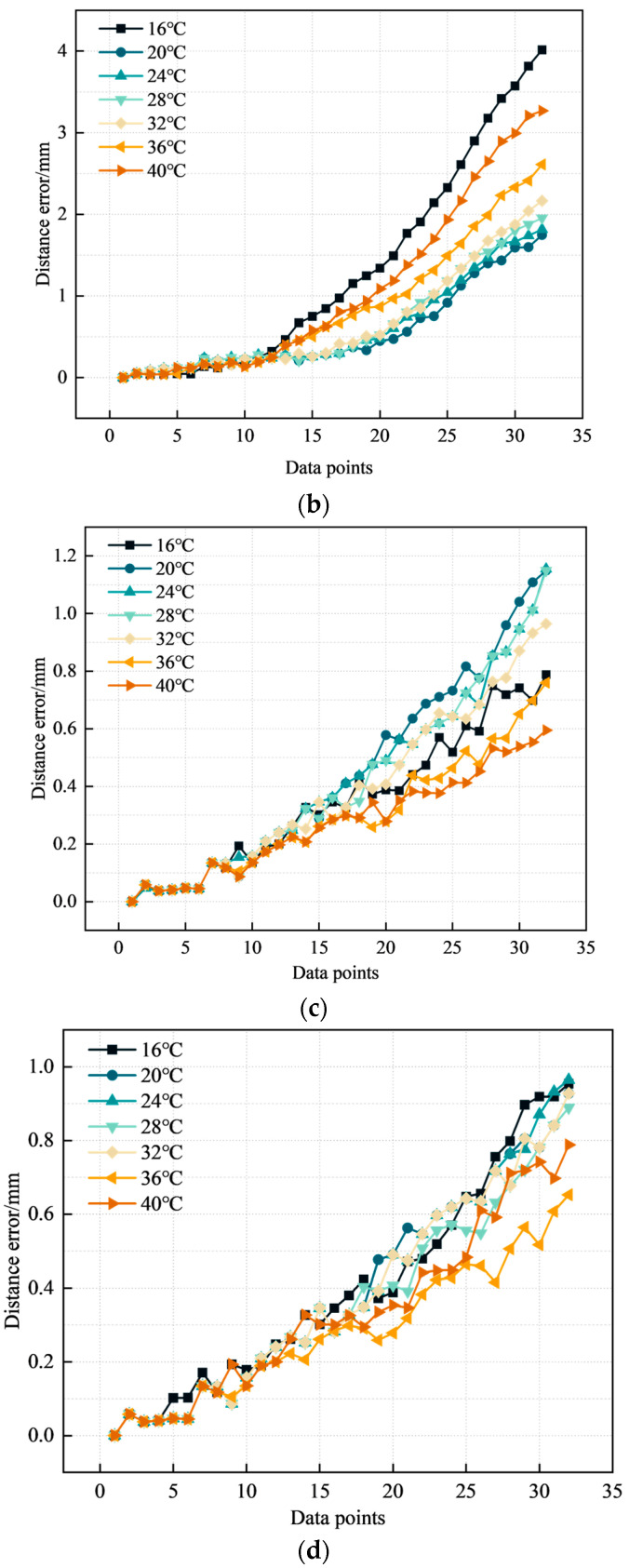
Catheter shape reconstruction results and errors in variable temperature environments. (**a**) Results of catheter shape reconstruction. (**b**) FBG distance error. (**c**) BP distance error. (**d**) PSO-BP distance error.

To verify the accuracy of catheter shape reconstruction due to temperature variations, we conducted a catheter shape reconstruction test in an environment with variable temperatures. The catheter was placed in a curvature groove with a curvature of 4.348 m^−1^, fixed to a curvature plate, and placed in a temperature control box with other equipment outside the box. The temperature range was set between 16 and 40 °C, varying at 4 °C intervals. After temperature stabilization, we recorded the FBG central wavelength and temperature values. We subsequently performed catheter shape reconstruction in BP and PSO-BP neural networks. Figure 11 represents catheter shape reconstruction after the neural network at different temperature conditions.

Figure 11a shows the results of the PSO-BP neural network catheter shape reconstruction at different temperatures. In the temperature range of 16–40 °C, the average distance error by the curve reconstruction algorithm is 0.82 mm and the maximum distance error is 4.01 mm. The maximum average distance error in the BP neural network is 0.37 mm and the maximum distance error is 1.15 mm. The average distance error in the PSO-BP neural network is 0.36 mm and the maximum distance error is 0.96 mm. Comparing the different methods shows that the PSO-BP neural network reconstructs the catheter shape with less error and better results. Furthermore, the temperature has a minor effect on the catheter shape measured by temperature compensation and the PSO-BP neural network.

In variable temperature environments, the average distance errors calculated by the curve reconstruction algorithm are all greater than the average distance errors calculated by the neural network. Firstly, the calculated curvature k in Section 3.1 is affected by parameters such as the distance from the outer to the middle core and the coefficient of photoelasticity. These parameters are provided by the manufacturer and do not represent actual values, so they affect the calculated curvature results. Secondly, when the multi-core optical fiber is integrated into the catheter, it is not strictly in the position of the central axis of the catheter, and only one end of the multi-core optical fiber and the catheter is fixed. When the catheter is bent, the multi-core optical fiber will deviate from the position of the central axis, resulting in calculation errors. Thirdly, the catheter is fixed to an aluminum alloy curvature plate, and although the temperature control box shows the specified temperature, the curvature plate does not reach the specified temperature. As a result, the catheter does not reach the specified temperature. Therefore, even if the wavelength shift is compensated for by the temperature, the reconstructed shape will be affected by the temperature. Even if the temperature change is minor, the reconstructed shape will show significant errors. In summary, our later work will focus on the sensing structure of the catheter to reduce temperature-induced errors.

## 5. Conclusions

In this paper, we present a PSO-BP neural network-based multi-core optical fiber sensing method for catheter shape measurement in interventional procedures. Firstly, we designed the catheter structure for the implanted optical fiber sensor to sense the catheter shape by integrating seven cores of optical fiber into the catheter. Secondly, we propose a PSO-BP neural network-based multi-core optical fiber sensing catheter shape reconstruction method to improve the accuracy of catheter shape-sensing and reduce errors caused by the characteristic parameters of optical fibers. We also studied a temperature compensation method to eliminate the influence of temperature changes on the FBG wavelength shift. Finally, to verify the effectiveness of the PSO-BP neural network-based multi-core optical fiber sensing catheter shape reconstruction method, we performed catheter shape reconstruction experiments in constant and variable temperature environments and compared and analyzed the errors of multi-core optical fiber sensing catheter shapes with different neural networks. Our experimental results show that the average and maximum distance errors of the PSO-BP neural network in reconstructing the shape under constant temperature conditions are 0.57 mm and 1.33 mm respectively. The average and maximum distance errors under variable temperature conditions are 0.36 mm and 0.96 mm. The PSO-BP neural network-based catheter shape-sensing method described in this paper can achieve accurate sensing of catheter shape. Future work will focus on catheter navigation in a patient’s vascular model and fuse multi-core optical fiber and electromagnetic sensors for locating catheter shape positions.

## Figures and Tables

**Table 1:** Adjustment results of neural network parameters.

Number of Hidden Layers	Hidden Size	Average Error/mm^−1^	Standard Deviation/mm^−1^	Training Time/s
1	3	0.4667	0.3966	4.29
1	6	0.4160	0.3386	4.31
1	9	0.4243	0.3132	4.60
1	10	0.4125	0.3357	4.79
1	20	0.4384	0.3545	5.18
1	50	0.4510	0.3722	6.30
1	80	0.4349	0.3603	8.91
1	100	1.6262	1.0784	9.75
2	3	0.6533	0.5031	4.55
2	6	0.4369	0.3557	4.99
2	9	0.4286	0.3602	5.67
2	20	0.7035	0.5444	8.58
2	50	2.5701	2.4314	12.60
2	100	2.6782	2.4266	22.15

## Data Availability

Not applicable.
